# Physiological and pathological neuronal connectivity in the living human brain based on intracranial EEG signals: the current state of research

**DOI:** 10.3389/fnetp.2023.1297345

**Published:** 2023-11-30

**Authors:** Yulia Novitskaya, Matthias Dümpelmann, Andreas Schulze-Bonhage

**Affiliations:** ^1^ Epilepsy Center, Department of Neurosurgery, Medical Center—University of Freiburg, Faculty of Medicine, University of Freiburg, Freiburg, Germany; ^2^ Department of Microsystems Engineering (IMTEK), University of Freiburg, Freiburg, Germany; ^3^ Center for Basics in NeuroModulation, Faculty of Medicine, University of Freiburg, Freiburg, Germany

**Keywords:** intracranial EEG, effective connectivity, functional connectivity, network, human brain, cortico-cortical evoked potentials

## Abstract

Over the past decades, studies of human brain networks have received growing attention as the assessment and modelling of connectivity in the brain is a topic of high impact with potential application in the understanding of human brain organization under both physiological as well as various pathological conditions. Under specific diagnostic settings, human neuronal signal can be obtained from intracranial EEG (iEEG) recording in epilepsy patients that allows gaining insight into the functional organisation of living human brain. There are two approaches to assess brain connectivity in the iEEG-based signal: evaluation of spontaneous neuronal oscillations during ongoing physiological and pathological brain activity, and analysis of the electrophysiological cortico-cortical neuronal responses, evoked by single pulse electrical stimulation (SPES). Both methods have their own advantages and limitations. The paper outlines available methodological approaches and provides an overview of current findings in studies of physiological and pathological human brain networks, based on intracranial EEG recordings.

## 1 Introduction

Over the past decades, the world neuroscience society became increasingly interested in human brain connectivity so that the yearly number of publications available under these keywords shows sustainable growth from several hundred in the early 2000s to several thousands in the last years. The attention shifts from a single brain region, traditionally considered a functional module, to interactions among distributed neuronal populations and brain areas. Modern neuroscience theories do not consider the brain as a number of fragmented components with a particular functional role, but rather emphasize the role of dynamically organized neuronal systems, probably underlying flexible cognitive operations and complex human behaviour (Bressler and Menon, 2010; Mill et al., 2017). This approach gave rise to novel concepts encouraging research in the field of network neuroscience that views the brain functioning in terms of dispersed but interconnected regions coordinating their activity in time- or frequency-related manner (Bassett and Sporns, 2017).

Brain connectivity can be described as structural (or neuroanatomical), functional, and effective connectivity. Structural connectivity refers to existing neuroanatomical links and can be evaluated over various neuroimaging modalities that provide comprehensive network maps of anatomical connections among neural elements. Although MRI-based tractography can identify fiber locations of white matter bundles (Grier et al., 2020), it is limited to major tracts and does not provide information about directionality of the connections. Functional MRI (fMRI) is a valuable non-invasive research tool for measuring and mapping brain activity with high spatial precision (up to 1,5–3 mm) in default state or during specific tasks. However, due to its low temporal resolution, limited by the haemodynamic response introducing a time delay of several seconds range between neuronal firing and changes of the BOLD signal (Harris et al., 2011; Drew, 2019; Amemiya et al., 2020), fMRI cannot be used for dynamic mapping of neural activity operating on a timescale of milliseconds. Non-invasive EEG and MEG, two connectivity methods with high temporal resolution in the required time range, are characterized, however, by relatively low spatial precision in the range of 4–5 cm as well as little or no sensitivity to activity in neuronal circuits located deeply under the scalp surface (Barkley and Baumgartner, 2003; Burle et al., 2015).

Under specific diagnostic settings, neural activity can be recorded directly from the living human brain by means of intracranial EEG. This method offers the opportunity to record neuronal signals precisely defined in space and time, also from deep brain structures not accessible for non-invasive EEG (Gonzalez-Martinez, 2016; Khoo et al., 2020). Here we review the method of invasive EEG and its application in modern connectivity research.

## 2 Measurement of intracerebral connectivity

### 2.1 Methodological approach of invasive brain research

Intracranial EEG (iEEG) signal in the living human brain can be recorded in tertiary epilepsy centres in patients with therapy resistant epilepsy as a part of presurgical evaluation. Presurgical epilepsy diagnostics is designed to delineate an epileptogenic focus that can be surgically removed in order to cure or substantially reduce seizures (Gonzalez-Martinez, 2016; Khoo et al., 2020). Due to very diverse localisation of epileptogenic focus, no other neurological condition allows the same opportunities for intracranial electrophysiological studies in neuroscience.

There are two most common methods for recording directly from the cortex in epilepsy patients: stereoelectroencephalography (SEEG) and subdural electrode recordings (SDEs) (Katz and Abel, 2019). SEEG requires insertion of intracerebral depth electrodes through drilled holes in the scull to stereotactically targeted cortical areas which are hypothesized to be involved in the seizure onset, propagation of epileptic activity or generation of seizure symptoms. SDEs are implanted through a craniotomy and, in case of strip electrodes, also through drilled holes. These two approaches can be combined, for example, for a dense coverage of the temporal lobe including anterior mesial structures. Whereas SDEs provide a dense coverage of the gyral surface, SEEG allows implantation of sulcal gray matter and deep brain structures, not accessible by SDEs. The obtained iEEG signal represents a two-dimensional view of cortical activity when recorded with SDEs and a three-dimensional view when recorded with SEEG. SEEG depth electrodes can be modified by adding microwires. Such combined (hybrid) electrodes contain a set of eight microwires protruding from the electrode tip that allows to simultaneously record intracranial EEG and single-unit activity, so called a hybrid SEEG (Miller et al., 2013; Nagahama et al., 2023). iEEG signal can be recorded at high sampling rates >2,000 Hz to cover high-frequency oscillations or even unit activities, and has a good signal-to-noise ratio with low artefact contamination (Ball et al., 2008; 2009).

Providing a great opportunity for human brain research, intracranial EEG studies have certain limitations. Although intracranial recording can be potentially performed in any brain region, the indication to undergo invasive EEG and the sites of electrode implantation are based exclusively on clinical reasons in order to localize the seizure onset zone, and cannot be justified by scientific interest only (Männlin et al., 2023). Therefore, the electrodes placement, their spatial orientation and implantation density may vary significantly among patients. For the same reason, invasive brain coverage is usually restricted by one or several cortical areas, forming a “tunnel view” and excluding large brain areas from the assessment of intracerebral communication in the same subject. Furthermore, interpretation of intracranial EEG findings needs to be done careful considering possible misinterpretation of the properties of interdependent neuronal circuits due to a large number of influencing factors, including also epileptogenicity and effect of antiseizure medication taken by patients. iEEG evaluation has to be performed by skilled EEG readers who are trained to distinguish between pathological and normal physiological neural activity that might appear to be epileptiform, for example, hippocampal theta waves (Lega et al., 2012), hippocampal ripples (Zhen et al., 2021), or sleep spindles (Gonzalez et al., 2022). Besides the factors mentioned above, the EEG findings can be influenced by recording technologies and filters incorporated in medical devices, since neuronal connectivity values have been reported to differ between depth electrodes and subdural electrodes (Sanz-Garcia et al., 2018; Bernabei et al., 2021), probably due to different patterns of spatial sampling.

### 2.2 Estimates of EEG-based brain connectivity

**FIGURE 1:**
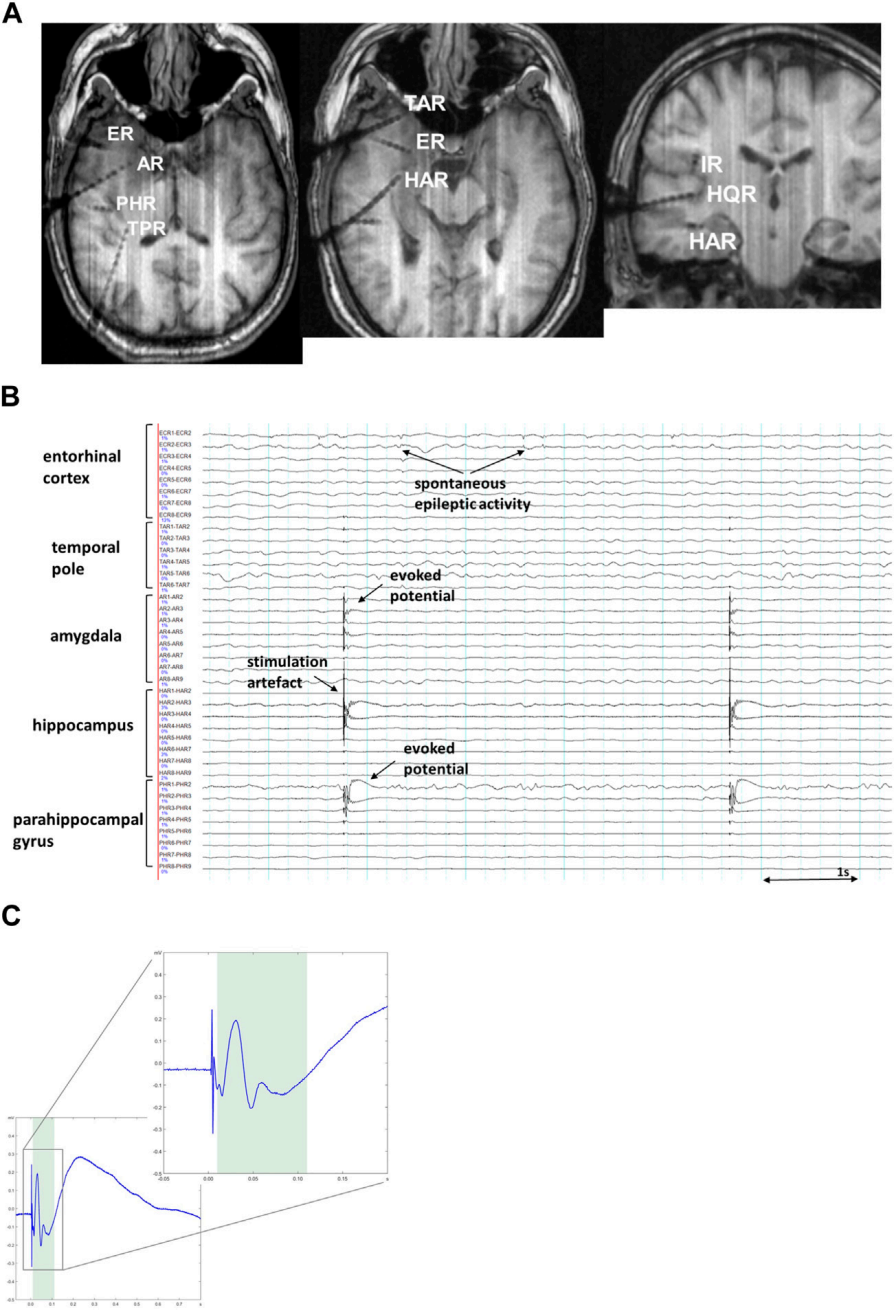
**(A)**. MRI imaging (T1-MPRAGE) in a representative patient showing intracranial positioning of depth electrodes in the right temporal lobe, adjacent insular cortex (IR) and Heschl’s gyrus (HQR). **(B)**. Exemplary SEEG snapshot showing cortico-cortical evoked potentials (CCEP), the most prominent on PHR1-2 (parahippocampal gyrus) and a smaller response on AR1-3 (amygdala) after stimulation on HAR1-2 (hippocampus). Sensitivity 70 μV/mm, low pass 120 Hz, high pass 1.6 Hz. **(C)**. A representative CCEP trace showing a typical curve with clearly visible sharp potential and a following slow-wave like discharge, corresponding to early N1- and late N2-components of CCEP waves respectively. The zoomed-in insert demonstrates the magnified early N1-component of the same CCEP trace. The shaded area indicates the time interval (10–110 ms) during which assessment of the early N1-component is typically made.

There are basically two approaches to assess brain connectivity in the EEG-based signal: evaluation of spontaneous neuronal oscillations during ongoing brain activity (Mayhew et al., 2013; Cantou et al., 2018) termed *functional* connectivity, and analysis of the electrophysiological neuronal responses, evoked by a SPES, single pulse electrical stimulation (Lacruz et al., 2007; David et al., 2010; Mandonnet et al., 2010; Keller et al., 2014; Kunieda et al., 2015; Krieg et al., 2017), so called *effective* connectivity. Measurements of functional connectivity estimate whether spatially disparate neurophysiological events appear to be temporally related (Friston, 1994; Daunizeau et al., 2011; Friston, 2011). Functional connectivity measurements do not require any direct intervention in the nervous system and can be implemented either in the absence of identifiable stimuli or in the context of performing a specific task. The SPES approach, on the contrary, uses active interference with the implanted cortical areas (Catenoix et al., 2005; Catenoix et al., 2011; David et al., 2013). In this setting, direct cortical stimulation at a low frequency of up to 5 Hz evokes electrophysiological responses (corticocortical evoked potentials, CCEP) at other intracranial recording sites (Figure 1). The SPES procedure has been shown to be safe for patients under the established protocol (Kobayashi et al., 2021). This renders cortical stimulation an important tool for mapping effective connectivity between brain regions which can be determined as quantification of the causal influence that one brain area may have over another (Friston, 1994; Friston, 2011; Trebaul et al., 2018).

Connectivity evaluation based on the analysis of spontaneous neuronal oscillations addresses various types of linear (Kramer et al., 2009) and non-linear (Lehnertz, 1999; Elger et al., 2000) relationships between the ongoing EEG dynamics recorded in regions of interest, allowing by that an assessment of functional connectivity, which is defined as “temporal correlation of a neurophysiological index measured in different brain areas” (Friston, 1994; Daunizeau et al., 2011; Friston, 2011). There are various methods operating with phase synchrony, a measure of consistency in phase difference, in a frequency domain between pairs of oscillatory signals across a set of observation, or purely with amplitude of the oscillations in order to quantify amplitude correlations independent of phase relations. Among measures of phase synchrony widely applied in neuroscience, some methods, such as phase slope index and phase locking value, are aimed to capture synchronized interactions between neuronal signals, yet limited to predominantly unidirectional interaction (Bastos and Schoffelen, 2016). Methods quantifying bidirectional interactions between two signals have also found broad application in neuroscience. These methods, such as Granger causality and alternative causality measures designed on its background, for example, directed transfer function and partial directed coherence, are based on the statistical dependencies between different neuronal signals estimated on the basis of multivariate linear models or cross-spectral densities. They provide information about bidirectional interactions, thus quantifying the directed influence of signal x on signal y and *vice versa* (Chen et al., 2006; Kaminski et al., 2016; Heyse et al., 2021).

Interpretation of estimated functional connectivity has to be performed with caution since there are several methodological issues that might influence the results (Friston et al., 2014; Bastos and Schoffelen, 2016). The main issue relates to the fact that EEG-recorded signals contain a poorly known mixture of signal-of-interest and background “noise” that might require a *post hoc* signal correction (Sommerlade et al., 2015). Another concern in interpretation of neuronal interactions is caused by volume conduction, a spatial spread of electromagnetic fields picked up by recording electrodes from multiple neuronal sources (Lopes da Silva, 2004). There are several strategies to overcome the influence of volume conduction in EEG signal: applying non-zero-time lags between the signals, or using connectivity metrics that functioning on the out-of-phase interaction, discarding the interactions that are at a phase difference of 0° (or 180°) (Bastos and Schoffelen, 2016). After all, observed connectivity patterns can be erroneously interpretated due to the fact that it is impossible to state whether an observed connection is a direct connection, or whether this connection is mediated through a third unobserved source.

Graph measures can be also applied for connectivity estimation, however, in case of iEEG, mainly as a measure of relatively closely spaced nodes (local topology) than long-range connections over the whole brain (global topology). These metrics nevertheless allow to rank the explored structures according to their degree of connectivity (Wilke et al., 2011; Gleichgerrcht et al., 2015; Hatlestad-Hall et al., 2021).

## 3 Effective brain connectivity

### 3.1 CCEP as a brain mapping tool

The first attempts to probe connectivity in the living human brain date back to 1980’s when bidirectional connections between human amygdala and hippocampus were stated through electrical stimulation in one structure and recording of the elicited response in another structure in the intracranial EEG (Buser and Bancaud, 1983). Nearly a decade later, single pulse electrical stimulation was applied to detect adjacent and remote cortical responses in the human temporal lobe, including cortical evoked potentials in the contralateral mesial temporal regions (Wilson et al., 1990; Wilson et al., 1991). In the early 2000s, several groups across the world established SPES-based methods to map cortical connectivity through cortico-cortical evoked potentials (Valentin et al., 2002; Matsumoto et al., 2004), considering cortical stimulation as an important tool for mapping effective connectivity between brain regions.

CCEP-based effective connectivity has been traced in numerous brain circuits, including language system (Matsumoto et al., 2004; David et al., 2013), motor system (Matsumoto et al., 2007; Kikuchi et al., 2012), frontal lobe network (Garell et al., 2013), temporal lobe and limbic network (Catenoix et al., 2005; Kubota et al., 2013; Takeyama et al., 2019; Novitskaya et al., 2020; Oane et al., 2020), parietal cortices (Togo et al., 2022) as well as auditory (Howard et al., 2000; Brugge et al., 2003) and visual (Sugiura et al., 2020) systems. The presence of CCEP in investigated brain regions is interpreted as functional tractography in comparison to anatomical fiber tractography derived from diffusion tensor imaging.

CCEPs frequently contain two major components: an early negative component (N1; <100 ms; Figure 1C) reflecting oligosynaptic connectivity in local cortical circuits (Matsumoto et al., 2004) as well as a late (N2; >100 ms) component generated via either cortico-cortical or cortico-subcortico-cortical polysynaptic pathways (Matsumoto et al., 2004; Matsumoto et al., 2007; Keller et al., 2014). The early N1-components are considered to reflect structural and effective connections between cortical regions and are used for brain mapping, whereas the late N2-components are considerably modulated by excitability of regions involved, such as an epileptogenic zone (Valentin et al., 2005). CCEPs have been demonstrated to have some variations in waveform and latency in different anatomical areas (Keller et al., 2011; Keller et al., 2014; Novitskaya et al., 2020). One study reported amplitude differences depending on hemispheric dominance in CCEPs obtained from temporoparietal areas (Kanno et al., 2018). The N1 peak latency has been demonstrated to correlate with the distance between stimulated and recorded areas, which may suggest the cortico-cortical propagation as a major cause of observed delays in the N1 response (Trebaul et al., 2018). The applied stimulation current is known to influence amplitude and spatial spread of CCEP (Kundu et al., 2020).

A recent work devoted to mapping large brain areas based on 774 445 cortico-cortical evoked potentials obtained from 780 patients with epilepsy revealed that the cortico-cortical axonal conduction delays between 57 investigated cortical areas were globally short with the median latency of 10.2 ms and associated to a median velocity of 3.9 m/s in a group of probands older than 15 years old (Lemaréchal et al., 2022). Axonal conduction delays were significantly larger in the group of subjects younger than 15 years, which corroborates that brain maturation increases the speed of brain dynamics (Lemaréchal et al., 2022; van Blooijs et al., 2023).

Changes in N1-components have been also shown in the context of sleep/wake cycle and the changes depended on the sleep stage. Compared with the awake state and, less prominent, with REM sleep, single-pulse stimulation during NREM sleep revealed increased connectivity and neuronal excitability which was expressed in the increased size of N1-waves and CCEP-related high-gamma activities, most noticeable in the frontal lobe (Usami et al., 2015). The finding suggests more intense neuronal activation occurred during NREM sleep than in the awake state. Increased cortical excitability during sleep regardless of epileptogenicity has been also confirmed in a recent CCEP-study (Arbune et al., 2020).

### 3.2 CCEP studies of the epileptogenic network

Cortical stimulation has been used for diagnostical purposes in epilepsy since more than 50 years. During invasive epilepsy evaluations, cortical stimulation in range of 25–50 Hz is applied to delineate eloquent (i.e. carrying out basic neurological functions) cortical regions and confirm epileptogenic cortex (Trébuchon and Chauvel, 2016). The SPES method that utilizes low frequency (up to 5 Hz) electrical stimulation has been also reported for the purpose of localizing the seizure onset zone and mapping the seizure propagation zone. In this case, besides the early CCEPs discussed above, late responses occurring later than 100 ms after stimulus (i.e. N2 component), as well as repetitive responses with longer lasting oscillations are linked to areas with changed (increased) excitability, such as an epileptogenic zone (Valentín et al., 2002; Valentín et al., 2005).

Several studies have reported changes in the morphology, latency and response rate of evoked potentials in epileptogenic brain regions (Rosenberg et al., 2009). Higher CCEP-amplitude have been shown in relation to seizure onset zone (Zhang et al., 2018; Hays et al., 2023) and seizure propagation zone (Lega et al., 2015). Large amplitude oscillations evoked through single-pulse electrical stimulation in clinically suspected epileptogenic areas have been recently proposed as an additional biomarker of the seizure onset zone (Smith et al., 2022).

Several studies have provided evidence that focal epilepsy arises from disordered neural connectivity in localized cortical regions that involves a concept of abnormal cortical networks with nodes and pathological connections also beyond the seizure onset zone. Epileptic networks have been found to include highly interconnected nodes with the highest number of outgoing connections (van Mierlo et al., 2013; Courtens et al., 2016) or ingoing connections (Li et al., 2016) in the seizure onset zone in patients with good postoperative outcome. These findings have been recently reassessed by means of early CCEP in epilepsy patients who underwent intracranial electrocorticography. The constructed effective networks revealed a high level of ingoing and outgoing connections, and a higher proportion of bidirectional connections in epileptogenic tissue, confirming that the epileptogenic tissue is densely connected with itself and, again, suggesting the SPES method as a valuable tool for localization of epileptogenic area (van Blooijs et al., 2018; Guo et al., 2020).

Focal epilepsy is frequently associated with a structural cortical lesion visible in brain MRI scans. Effective connectivity that seems to reflect anatomical oligosynaptic pathways in local cortical circuits can be potentially altered by any structural abnormalities in the assessed regions. Epileptogenic lesions are histologically various and can be roughly attributed to either local cellular loss (e.g., hippocampal sclerosis, posttraumatic lesion) or cortical malformations (e.g., focal cortical dysplasia, tuberous sclerosis, tumors). Influence of structural brain abnormalities on CCEP properties has not been systemically explored yet. Electrophysiological study *in vitro* using hippocampal slices obtained from epileptic patients who had undergone epilepsy surgery did not reveal any effect on stimulus-evoked action potential in slices with mesiotemporal sclerosis compared to the hippocampal samples without a structural lesion (Knowles et al., 1992). Nevertheless, effects of electrical stimulation might be different on systemic levels. A single observation of Catenoix et al. (2011) provided evidence that severe hippocampal atrophy might be associated with disappearance of physiological CCEPs, suggesting that cellular loss could result in the degeneration of some hippocampal projections. A recent work which assessed hippocampal fast ripples evoked by parahippocampal SPES indirectly supports this assumption as it has been revealed that probability to evoke fast ripples decreased with the severity of hippocampal sclerosis in the areas CA2-3 but increased in the subiculum (Tóth et al., 2021).

Regarding malformational lesions such as a focal cortical dysplasia (FCD), a single report is available that demonstrates a difference in the amplitude of the SPES-responses with larger responses in patients with FCD type I compared to FCD type II (Shahabi et al., 2021). Another recent SPES-study in epilepsy patients with nodular heterotopias, a brain malformation of cortical development, has revealed that nodular heterotopias have widespread CCEP-based connectivity with the overlying cortex but also with distant cortical regions and other nodules (Boulogne et al., 2022).

## 4 Functional brain connectivity

### 4.1 Measures of “information flow”

Evaluation of spontaneous neuronal oscillations during ongoing brain activity does not require any direct intervention in the nervous system and can be implemented either in the absence of identifiable stimulus or during task performance as well as in the context of specific brain activity such as sleep or epileptic seizure. Neural activity evaluated as statistical dependencies among time series can be termed “Neural Information Flow,” meaning how much information is transferred between regions within the nervous system, the process which might underlie communicational processes within the brain (Dimitrov et al., 2011). Despite the promising application value, only few published works involve assessment of functional connectivity based on the iEEG signal, most likely due to methodological issues discussed above (Section 2.2).

Intracerebral functional connectivity has been mostly investigated within auditive and language networks. Regarding auditive perception, the timing and distribution of left perisylvian electrophysiological activity has been analysed using the Granger causality method in a single patient during a speech processing task and revealed a widespread activation over all temporal structures involved in the ventral speech processing pathway (Gow et al., 2009). In another study of emotional musical perception, information flow from the amygdala to the orbitofrontal cortex and from the amygdala to the auditory cortex was considerable and reliable across seven subjects, confirming the involvement of amygdala into emotional processing (Omigie et al., 2015). A functional connectivity study in a lexical selection task demonstrated that the Broca’s area was more active and also exhibited more local network interactions with posterior temporal cortex during the task (Wang et al., 2021).

Several recent studies addressed the issue of verification the results of functional connectivity methods by comparing them with CCEP networks. Hebbink et al. (2019) compared the overlap in connectivity between functional methods (cross-correlation and Granger causality) and the SPES-induced network in order to assess their ability to reveal well-known anatomical connections in the language circuit. Crocker et al. (2021) investigated effective and functional connectivity measures across numerous frontal and temporal sites during resting state. Both studies reported a divergence between effective and functional networks *in vivo*, suggesting unlike the evoked connectivity that revealed to be stable under different conditions, functional connectivity can be a dynamic process in spatial and temporal scales and probably vary state-dependently.

### 4.2 Functional connectivity in the epileptogenic network

Intracranial EEG is aimed to delineate the epileptogenic area, and the seizure onset zone is traditionally defined visually by treating epileptologists that can be sometimes very challenging. In order to support clinical iEEG interpretation, a computer-assisted approach named Epileptogenicity Index has been proposed (Bartolomei et al., 2008). The method was designed to quantify the level of involvement of each brain region in the ictal onset, and was based on a spectral analysis of the iEEG signal, emphasizing activity in the high frequency bands. In the following years, different computer-assisted signal analysis methods have been introduced and, when compared, showed highly discordant results depending on the specific seizure pattern (Andrzejak et al., 2015). That promoted research aimed to delineate the seizure onset zone based on its connectivity.

Focal epilepsy is considered to result from disordered local hyperexcitability and connectivity, originating from “driver” regions (Bui et al., 2015; Hebbink et al., 2017). In the last decade, numerous connectivity measures have been applied to study the epileptogenic network, including directed transfer function, Granger causality, linear and non-linear correlation, imaginary coherence, partial directed coherence and cross-frequency directionality, in time or time-frequency domains (Lagarde et al., 2022). Graph-theory measures have been also used in order to estimate the level of involvement of each brain region (i.e., graph node) within the epileptogenic network (Panzica et al., 2013; Gleichgerrcht et al., 2015; Ren et al., 2019; Hatlestad-Hall et al., 2021). The results of the studies suggest higher functional connectivity in brain areas located in the epileptogenic zone with gradual decrease in the connectivity level in propagation zone, irritative zones, and zones, not involved in the seizure generation (Lagarde et al., 2018; Goodale et al., 2020; Narasimhan et al., 2020). Moreover, epileptogenicity has been shown to be in causal relationships with the surrounding neuronal populations (Sabesan et al., 2009; Wang et al., 2017). From the clinical point of view, causality measures are interesting in order to define the network nodes with the largest sum of outgoing links from a given brain region which indicates the regions with higher outflow as correlate to the seizure onset zone (van Mierlo et al., 2013; Courtens et al., 2016). Changes in the pattern of functional connectivity have been barely studied in the context of cortical lesions. Focal cortical dysplasia, a brain malformation associated with intrinsic epileptogenicity, has been shown to be characterized by abnormal outgoing connectivity in comparison with the other examined areas (Varotto et al., 2012).

Functional connectivity studies suggest that epileptogenic networks exhibit aberrant dynamics not only at the time of seizure onset, but also during interictal seizure-free periods. A causality-based analysis has revealed that persistence of inflows and outflows of high frequency activity was a good indicator of the seizure onset zone, also observed during interictal periods (Korzeniewska et al., 2014). Interictal connectivity directed towards the assumed seizure onset zone gave rise to the interictal suppression hypothesis that suggests that epileptogenic zones have an increased inward connectivity which could relate to interictal suppression of epileptiform activity as a control mechanism during seizure-free periods (Vlachos et al., 2017; Narasimhan et al., 2020; Jiang et al., 2022; Johnson et al., 2023).

## 5 Challenges and future directions

iEEG is a valuable tool for neuroscience studies in the living human brain, however, its scientific implementation can be challenging. Firstly, invasive EEG recording is restricted to investigations in epilepsy patients, and can be performed only in clinically relevant cortical regions in a given patient limiting the area of investigation. This issue can be overcome by collecting iEEG data from many patients in multiple tertiary epilepsy centers and fusing them to a large database (for example, see *f-tract.eu*; Lemaréchal et al., 2022) which allows for sampling information across extended brain. Yet, the inhomogeneity of the data should be considered as the iEEG recordings are acquired from patients of different sex, age and neurological conditions. Strategies of electrode placement and iEEG acquisition can vary substantially between epilepsy centers. Also, SEEG cortical stimulation practices have been recently shown to be very different across hospitals (Cockle et al., 2023). Additionally, epilepsy patients often have cognitive impairments and psychiatric comorbidities which may affect memory formation, affective processing and other fundamental functions of human cognition. Depending on the degree of such impairments, this may limit the extrapolation of iEEG-based research results to the healthy population.

iEEG-based connectivity assessment has a promising application in mapping epileptogenic networks. Nowadays, the identification of the SOZ during presurgical evaluation is a manual, time-consuming process which operates with large amounts of disparate neural data and has a basically unexplored reliability (Flanary et al., 2023). Although a number of automated approaches using machine and deep learning have been applied to identify SOZ, the visual iEEG reading by epileptologists remains the gold diagnostic standard worldwide. Whereas an unsupervised automated iEEG exploration is still not the matter of the nearest future, developing neural biomarkers that have a supportive value for visual SOZ search is encouraging. So far, a few retrospective SEEG studies have found structural and functional connectivity to be predictive markers for postsurgical outcomes (Shamas et al., 2022; Stone et al., 2022; Matarrese et al., 2023; Sinha et al., 2023; Xu et al., 2023). The assessment of neural connectivity may thus in the future becomes a tool of presurgical evaluation for better patient selection, and for improved strategies of tailored brain resection or neurostimulation of the epileptogenic focus (Khambhati et al., 2021). Beyond the field of epileptology, iEEG-based studies of human brain connectivity may gain an insight into the brain organization under different physiological and pathological conditions such as sustained wakefulness, NREM sleep, mental disorders, pain, cognitive impairments, or cognitive performance. Intracranially recorded brain activity can be used to identify circuit-level electrophysiological correlates of neurological and psychiatric symptoms in order to modulate the circuits over targeted electrical stimulation to disrupt the symptoms. This approach has been successfully applied since decades by the example of deep brain stimulation in thalamus for treatment of movement disorders, most commonly Parkinson disease (Lozano et al., 2019). The same strategy can be used for targeting electrophysiological features which correspond to affective, cognitive or sensory symptoms. This can provide a starting point for developing more effective neuromodulation interventions and extending treatment options in psychiatry or pain management (Bormann et al., 2021).

Besides spontaneous neural activity, intracranial EEG recording can be performed in association with stimuli of different modalities, allowing investigation of human event-related potentials. This found application in studies of cognitive or affective activity involved in processing the stimulus or preparing an action (Fernández et al., 1999; Helfrich and Knight, 2019). Event-related potentials recorded in the substructures of the human medial temporal lobe have been shown to differentiate in the latency and localization in a paradigm of language comprehension (Meyer et al., 2005) and a paradigm of face recognition (Dietl et al., 2005), thus suggesting functionally distinct aspects of language integration and associative semantic memory processes. Recordings of single unit activity can be beneficial for such kind of studies (Lakretz et al., 2021).

Lastly, the assessment of electrophysiological connectivity can add an extra-layer of information onto structural networks derived from brain topography methods such as DWI, or supplement functional fMRI-based networks in the context of task performance or other psychological processes. Future studies may integrate data from the iEEG-based connectivity research with the MRI tractography methods to determine the precise relationship between cognition and observed structural networks.
