# Supplementary material for: Physiological and pathological neuronal connectivity in the living human brain based on intracranial EEG signals: the current state of research
Source: Front Netw Physiol. 2023 Nov 30;3:1297345. doi: 10.3389/fnetp.2023.1297345 (PMC10723837; doi:10.3389/fnetp.2023.1297345)
Supplement: Supplementary file 1 [file DataSheet1.docx]

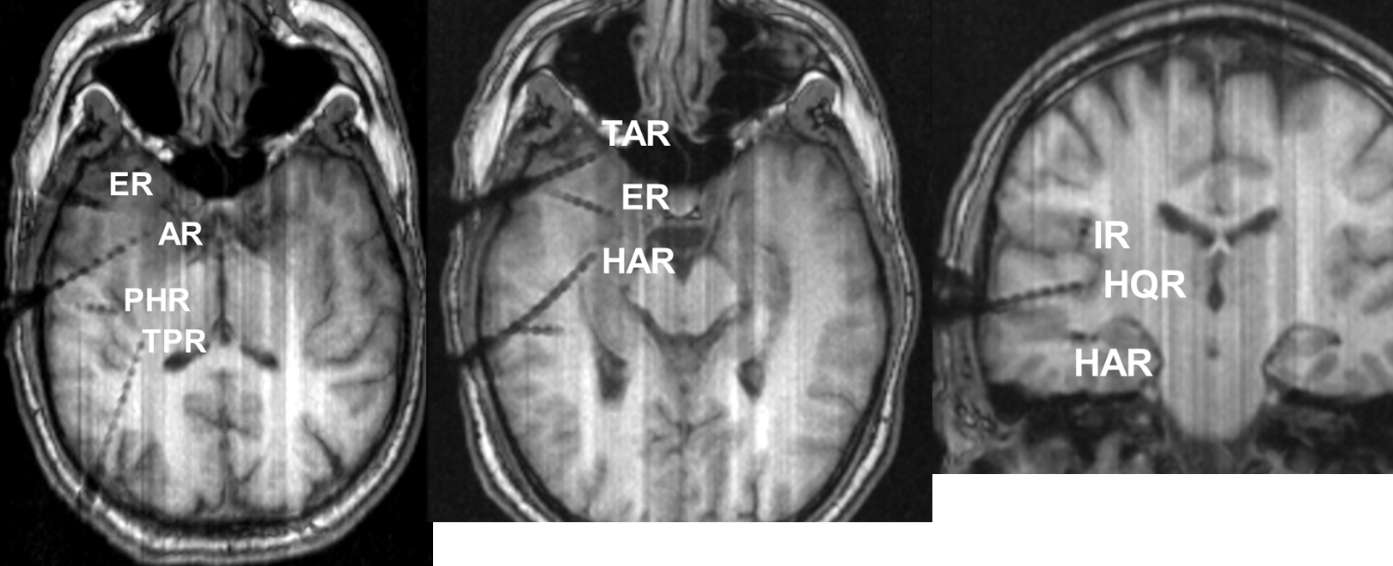


B.


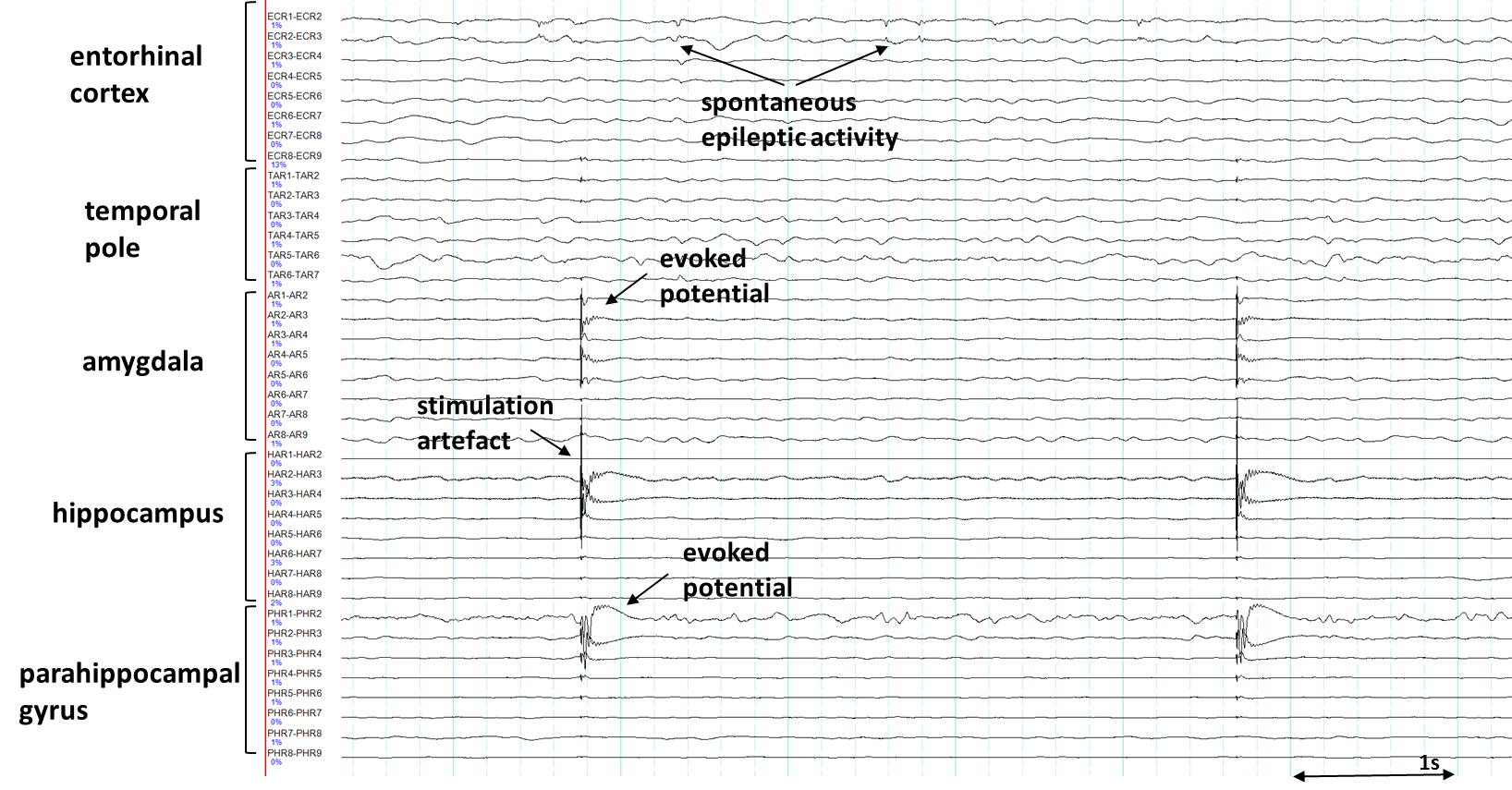


C.


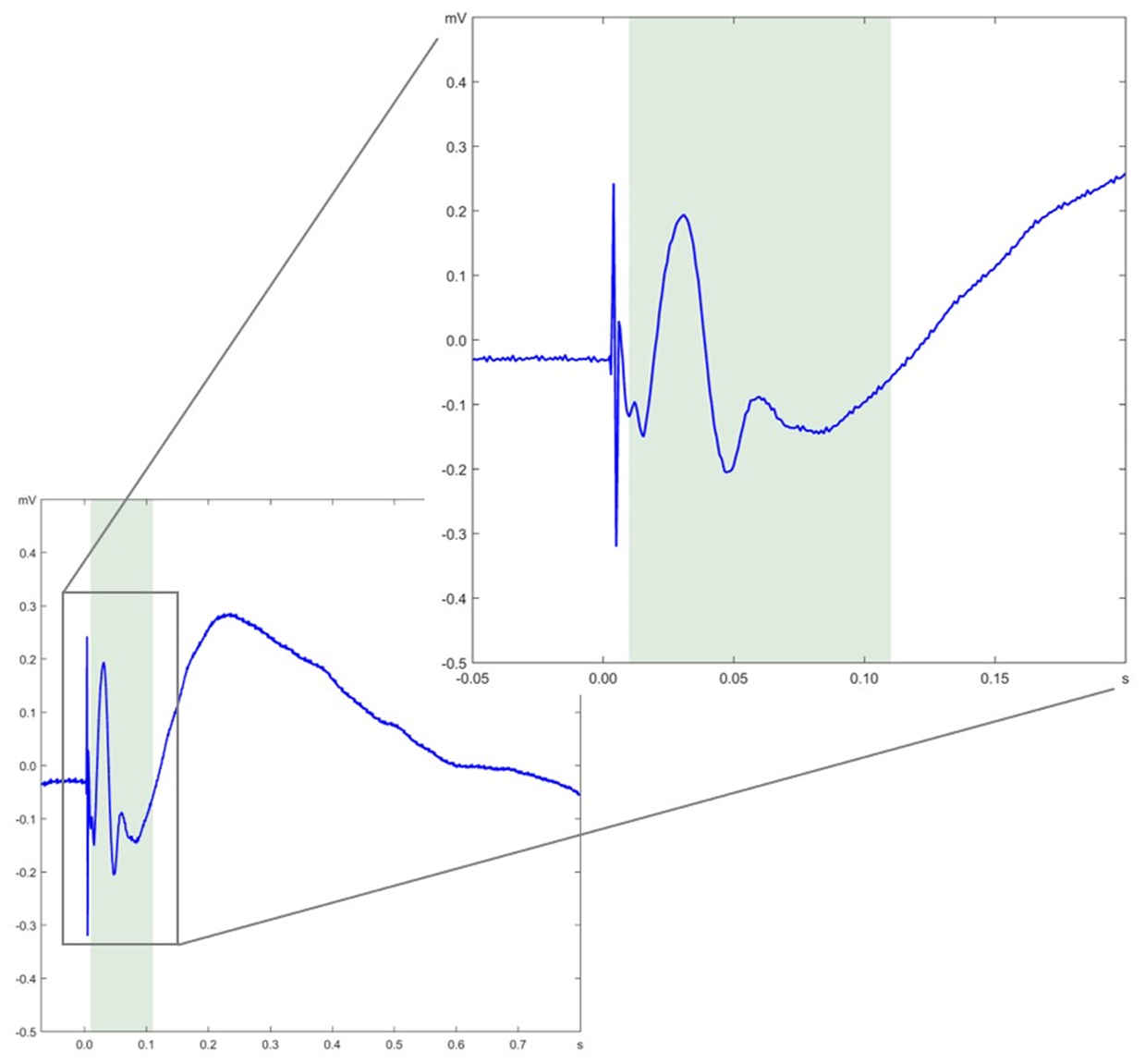


**Fig. 1.**

1. MRI imaging (T1-MPRAGE) in a representative patient showing intracranial positioning of depth electrodes in the right temporal lobe, adjacent insular cortex (IR) and Heschl's gyrus (HQR).
2. Exemplary SEEG snapshot showing cortico-cortical evoked potentials (CCEP), the most prominent on PHR1-2 (parahippocampal gyrus) and a smaller response on AR1-3 (amygdala) after stimulation on HAR1-2 (hippocampus). Sensitivity 70µV/mm, low pass 120 Hz, high pass 1,6 Hz.
3. A representative CCEP trace showing a typical curve with clearly visible sharp potential

and a following slow-wave like discharge, corresponding to early N1- and late N2-components of CCEP waves respectively. The zoomed-in insert demonstrates the magnified early N1-component of the same CCEP trace. The shaded area indicates the time interval (10 to 110 msec) during which assessment of the early N1-component is typically made.
